# Agreement of 3d-ssfp and echocardiography for aortic root dimensions

**DOI:** 10.1186/1532-429X-13-S1-P221

**Published:** 2011-02-02

**Authors:** Puneet Bhatla, Rowan Walsh, Elena Kwon, James Nielsen

**Affiliations:** 1 Mount Sinai, New York, NY, USA

## Background

Respiratory navigated, ECG-gated, 3D-isotropic steady state free precession (3D-SSFP) is increasingly utilized in pediatric aortopathy. This is particularly true in adolescents as acoustic windows become limited. The agreement between echocardiography (ECHO) and 3D-SSFP obtained measurements could be influence by imaging plane, timing (systole versus diastole) and image quality. With 3D-SSFP the image quality is generally superior when acquisition is timed to diastole. As diastolic measurements are utilized as standard practice in adult aortopathy decision-making, whereas systolic diameters are standard in pediatric practice, examining the agreement between diastolic 3D-SSFP and ECHO (both systole and diastole) has merit. This study examined this question retrospectively.

## Methods

Echocardiographic and CMR data from 21 consecutive subjects referred for CMR, with and without heart disease were retrospectively reviewed. Subjects with >6 months between examinations (N=1) or unavailable ECHO data (N=5) were excluded. A standard 3D-SSFP sequence was utilized. A single, experienced observer used multiplanar reformatting to define a CMR plane that mirrored a standard parasternal long-axis ECHO plane. The maximum inner diameters at three levels (aortic annulus, root and sinotubular junction (STJ)) were measured offline from 3D-SSFP and ECHO imaging data and analyzed by Bland-Altman method of agreement.

## Results

The median age was 15.3 years (range 8-26 years). The median (range) for the diameters was 2.34 (1.86-3.35), 3.18 (2.59-4.18) and 2.48 (2.09-3.65) cm for annulus, root and STJ, respectively. Table 1.

**Table 1:** 

Measurement site (timing)	N	r	Mean Difference (cm)	Limits of Agreement (cm)
Annulus (systole)	15	0.91	0.082	-0.28 to 0.44
Root (systole)	15	0.96	-0.030	-0.28 to 0.21
STJ (systole)	15	0.93	-0.240	-0.33 to 0.28
Total (systole)	45	0.95	0.009	-0.31 to 0.33
Root (diastole)	15	0.89	0.031	-0.40 to 0.46

## Conclusions

Given the current definition of rapid progression as >5mm per year, acceptable agreement was found for 3D-SSFP versus ECHO in this retrospective cohort with a mean difference near zero and tight limits of agreement. Careful attention to matching the imaging plane utilizing 3D multiplanar reformatting is key to maximizing the agreement between the two imaging modalities.

